# Association of Malnutrition, Inflammation, and Residual Kidney Clearance in Peritoneal Dialysis: A Cross‐Sectional Analysis

**DOI:** 10.1155/ijne/1300185

**Published:** 2026-08-13

**Authors:** Wankawee Jeerangsapasuk, Chayaporn Wichitkul, Thanarak Boonkerd, Siriporn Wisettorn, Aphichat Chatkrailert

**Affiliations:** ^1^ Division of Nephrology, Department of Internal Medicine, Faculty of Medicine, Thammasat University, Pathumthani, Thailand, tu.ac.th; ^2^ 60th Anniversary HRH Maha Chakri Sirindhorn Hemodialysis Center, Thammasat University Hospital, Pathumthani, Thailand, tu.ac.th

**Keywords:** anemia, erythropoietin resistance, Interleukin-6 (IL-6), malnutrition, malnutrition inflammation Score (MIS), peritoneal dialysis (PD), residual kidney clearance (Kr)

## Abstract

**Background:**

Malnutrition and systemic inflammation are common and interrelated complications in patients undergoing peritoneal dialysis (PD), contributing to poor clinical outcomes. Residual kidney clearance (Kr) may play a protective role in this context, while erythropoietin resistance index (ERI) reflects anemia management challenges influenced by inflammation. This study aimed to investigate the associations of nutritional and inflammatory markers with Kr and ERI in PD patients.

**Methods:**

We conducted a cross‐sectional study of 47 stable adult PD patients. Nutritional and inflammatory indicators, including Malnutrition Inflammation Score (MIS), serum albumin, and Interleukin‐6 (IL‐6), were assessed alongside Kr and ERI. Linear regression analysis was used to evaluate associations, and Spearman correlation with bootstrapped 95% confidence intervals determined relationships among clinical variables.

**Results:**

The mean age was 62.2 ± 15.5 years; 53.2% were male. The mean MIS was 5.7 ± 2.5, serum albumin 3.29 ± 0.5 g/dL. IL‐6 was positively associated with MIS (*β* = 0.684, *p* = 0.037). Kr was significantly inversely associated with MIS (*β* = −0.585, *p* = 0.040) and positively associated with serum albumin (*β* = 0.152, *p* = 0.018). Spearman analysis confirmed significant correlations between Kr and both MIS (*r* = −0.358, *p* = 0.008) and albumin (*r* = 0.343, *p* = 0.012), and between IL‐6 and MIS (*r* = 0.390, *p* = 0.004). ERI showed no significant correlations with MIS, albumin, IL‐6, or Kr.

**Conclusion:**

MIS was significantly associated with IL‐6, highlighting the close relationship between malnutrition and systemic inflammation in patients undergoing PD. Higher Kr was associated with a more favorable nutritional inflammatory profile, whereas no significant associations were observed for ERI.

## 1. Introduction

Chronic kidney disease (CKD) represents a significant and escalating global health challenge, with an increasing number of patients requiring kidney replacement therapies such as peritoneal dialysis (PD) [1, 2]. Malnutrition is highly prevalent among individuals with CKD and end‐stage kidney disease (ESKD), often aggravated by chronic inflammation and metabolic disturbances [3]. Suboptimal nutritional status has been consistently associated with adverse clinical outcomes, including increased rates of hospitalization and mortality [4–6]. Protein‐energy wasting (PEW), a syndrome characterized by the concurrent presence of malnutrition and systemic inflammation, is commonly observed in this population and has been strongly linked to a poor prognosis [7, 8]. The Malnutrition Inflammation Score (MIS), a validated composite tool, has been widely employed to assess both nutritional status and inflammatory burden in dialysis patients [9].

Chronic systemic inflammation is highly prevalent in patients with ESKD and is increasingly recognized as a key contributor to adverse clinical outcomes. Patients receiving dialysis frequently exhibit persistently elevated inflammatory biomarkers such as C‐reactive protein and Interleukin‐6 (IL‐6), reflecting a state of low‐grade but sustained inflammation associated with cardiovascular morbidity and mortality [10]. In PD populations, inflammation may originate from both systemic and local peritoneal sources; however, accumulating evidence suggests that systemic inflammatory activity, particularly elevated plasma IL‐6, plays a more prominent role in predicting mortality than local peritoneal inflammation [11]. These observations support the concept that systemic inflammation represents a central pathophysiological pathway influencing nutritional status, cardiovascular risk, and survival in patients with ESKD undergoing dialysis [12].

Residual kidney clearance (Kr) is increasingly recognized as a critical determinant of nutritional and metabolic homeostasis in ESKD patients [13]. Kr has been associated with enhanced uremic toxin clearance [14], reduced inflammation [15, 16], and improved volume control [17], all of which may contribute to a more favorable nutritional profile. Because Kr is associated with mortality and other adverse outcomes, ISPD guidelines recommend monitoring Kr every six months and emphasize the importance of preserving Kr whenever possible [18, 19]. However, the degree to which Kr is associated with systemic inflammation and nutritional markers in the PD population remains inadequately explored.

Anemia is another common and multifaceted complication of CKD and ESKD, contributing substantially to reduced quality of life and increased morbidity [20]. Although erythropoiesis‐stimulating agents (ESAs) remain central to anemia management in these patients, their efficacy is often limited by erythropoietin resistance, which necessitates higher ESA doses to achieve target hemoglobin (Hb) concentrations [21]. The pathophysiology of erythropoietin resistance is multifactorial, involving inadequate dialysis, iron deficiency, malnutrition, and chronic systemic inflammation [22]. Among proinflammatory cytokines, IL‐6 has been implicated in both systemic inflammation and ESA hyporesponsiveness [23–25]. This resistance has been independently associated with increased risks of hospitalization and mortality [26, 27], underscoring the importance of identifying modifiable contributors to optimize ESA responsiveness and clinical outcomes.

Although previous studies have reported associations between Kr and mortality, dialysis adequacy, or individual nutritional and inflammatory markers, the interrelationships among MIS, serum albumin, IL‐6, ERI, and Kr in PD patients remain less well characterized. This study aimed to evaluate the associations between MIS, serum albumin, IL‐6, Kr, and ERI in patients undergoing PD.

## 2. Materials and Methods

### 2.1. Study Design and Setting

This cross‐sectional study was conducted at the adult PD clinic between 1 December 2022 and 28 February 2023. Eligible participants were randomly selected during routine outpatient visits. Ethics were approved by The Human Research Ethics Committee of Thammasat University (MTU‐EC‐IM‐0‐096/64). Informed consent was obtained from all individual participants before enrollment. This study was reported in accordance with the Strengthening the Reporting of Observational Studies in Epidemiology (STROBE) guidelines [28].

### 2.2. Participants

Inclusion criteria were adults (≥ 18 years) with ESKD undergoing PD for at least three months, receiving ESA at a stable dose (no ESA adjustment for the prior three months), and with transferrin saturation (TSAT) > 20% and serum ferritin > 100 ng/mL. Only short‐acting ESAs were used across the cohort, minimizing pharmacokinetic variation. Patients were excluded if they had experienced acute illness within the past two months, had anemia from nonkidney hematological causes, or had received a blood transfusion or had overt blood loss within the previous two months. Individuals with incomplete clinical or laboratory data were also excluded.

### 2.3. Variables and Data Collection

Demographic information, including age and sex, was recorded. Laboratory parameters collected included blood urea nitrogen (BUN), serum creatinine, Hb, parathyroid hormone (PTH), calcium, and phosphate. Nutritional markers, including serum albumin and total iron‐binding capacity (TIBC), were obtained from routine blood tests. IL‐6 was measured using the Elecsys IL‐6 assay (Roche Diagnostic, Thailand). The lower limit of detection was 1.5–5000 pg/mL, and the intraassay coefficient of variation was 0.6%–4.1%. Dialysis adequacy and protein intake were assessed using total weekly Kt/V, protein catabolic rate (PCR), and normalized protein catabolic rate (nPCR), all obtained from the most recent results within three months prior to data collection. ESA dose was converted to the ERI, calculated as follows: ERI = weekly ESA dose (units)/[body weight (kg) × Hb (g/dL)]. The MIS score, used to assess nutritional and inflammatory status, a validated tool composed of 10 components, each scored from 0 (*normal*) to 3 (*severely abnormal*), based on medical history, physical examination, and laboratory data, yielding a total score ranging from 0 to 30 [9]. Data for MIS scoring included information on recent weight changes, dietary intake, gastrointestinal symptoms, functional capacity, and comorbidities, collected through structured interviews and physical examinations performed by trained personnel. Kr was calculated using a 24‐h urine collection obtained at home following standardized instructions. Urinary urea clearance was determined from the 24‐h urine volume and urine urea concentration using standard clearance equations as the ratio of urinary urea excretion (urine urea concentration × urine volume) to serum urea concentration. The urea distribution volume (V) was estimated using the Watson formula. Clearance values were normalized to body surface area (BSA) and expressed as mL/min/1.73 m^2^. Body weight was measured using a SECA 674 digital scale. Height was measured with a stadiometer or arm span (if patients were unable to stand). Body mass index (BMI) was then calculated and recorded.

### 2.4. Study Outcomes

The primary outcome was to examine the association between MIS and IL‐6 levels. Secondary outcomes included associations between serum albumin and IL‐6, nutritional surrogate markers (MIS and serum albumin) and ERI, and nutritional surrogate markers and Kr, and exploratory analyses of clinical and biochemical factors associated with IL‐6, ERI, and Kr.

### 2.5. Study Size

The study size was calculated based on the primary objective, which was to assess the correlation between MIS and IL‐6 levels in PD patients. Using a correlation‐based sample size calculation, we assumed an expected correlation coefficient (*r*) of 0.4, with a significance level (*α*) of 0.05 and statistical power of 80%. The sample size was estimated using the formula:

(1) n=Z12−α/+Z1−β0.5×ln11+r/−r2+3.

Based on these parameters, a minimum of 47 participants was required to detect a moderate correlation between MIS and IL‐6.

### 2.6. Bias

To minimize selection bias, sample size estimation was calculated, and eligible participants were randomly selected. Strict adherence to predefined inclusion and exclusion criteria helped reduce the risk of enrollment and data entry errors. To ensure consistency and minimize measurement bias, MIS assessments and other measurements were performed by trained nursing staff using standardized procedures and calibrated equipment.

### 2.7. Quantitative Variables and Statistical Methods

Statistical analyses were performed using STATA Version 16. Descriptive statistics were used to summarize patient characteristics. Continuous variables were expressed as mean ± standard deviation (SD) for normally distributed data or median with interquartile range (IQR) for nonnormally distributed data. Categorical variables were presented as frequencies and percentages. To evaluate associations between nutritional and inflammatory markers and study outcomes, ERI, IL‐6, and Kr were categorized into quartiles (Q1–Q4). Because several variables, including MIS and Kr, were not normally distributed, nonparametric methods were applied where appropriate. Linear regression models were constructed with MIS or serum albumin as continuous outcome variables, while quartiles of IL‐6, Kr, and ERI were entered as ordinal predictors. Spearman’s rank correlation coefficient was used to examine associations between clinical, biochemical, and dialysis‐related variables and ERI, IL‐6, and Kr. A nonparametric bootstrapping approach was applied to estimate 95% confidence intervals (CIs) for each correlation coefficient. Specifically, we generated 1000 resampled estimates of the Spearman rho (*r*) statistic. This method offers a robust, assumption‐free estimation of variability around the correlation coefficient regarding small sample sizes or skewed distributions. Regression results and correlation coefficients were reported with 95% CIs. Scatterplots were generated to visually illustrate the relationships between Kr and nutritional‐inflammatory markers. A *p* value < 0.05 was considered statistically significant.

## 3. Results

Patients in our PD clinic were assessed for eligibility and 51 of them met our inclusion criteria. However, four of them did not have IL‐6 result; these participants were excluded from the analysis.

A total of 47 PD patients were included. The mean age was 62.2 ± 15.5 years, and 53.2% were male. The mean Hb was 10.0 ± 1.6 g/dL, serum albumin was 3.29 ± 0.5 g/dL, and the mean MIS was 5.7 ± 2.5. More than half of the participants (57.5%) were at high risk of malnutrition (MIS ≥ 6), and 61.7% had serum albumin < 3.5 g/dL. The median IL‐6 concentration was 9.23 pg/mL (IQR 6.53–21.09), Kr was 0.07 mL/min/1.73 m^2^ (IQR 0–0.41), and ERI was 8.28 (IQR 5.56–14.18). The mean total weekly Kt/V was 1.87 ± 0.3. Data are shown in Table 1.

**TABLE 1:** Baseline characteristics.

Patients’ characteristic	Value (*n* = 47)
Age (years), mean ± SD	62.2 ± 15.5
Male gender, *n* (%)	25 (53.2%)

*Blood tests*	
Blood urea nitrogen (mg/dL), mean ± SD	56.0 ± 25.2
Creatinine (mg/dL), mean ± SD	10.6 ± 4.3
Hemoglobin (g/dL), mean ± SD	10.0 ± 1.6
Parathyroid hormone (pg/mL), median (IQR)	364 (195, 573)
Calcium (mg/dL), mean ± SD	9.3 ± 0.8
Phosphate (mg/dL), mean ± SD	4.5 ± 1.5

*Peritoneal dialysis adequacy and protein catabolic rate*	
Total weekly Kt/V (), mean ± SD	1.87 ± 0.3
Residual kidney clearance: Kr (mL/min/1.73 m^2^), median (IQR)	0.07 (0, 0.41)
Protein catabolic rate(g/day), mean ± SD	46.7 ± 12
Normalized protein catabolic rate (g/kg/day), mean ± SD	0.83 ± 0.3

*Malnutrition and inflammation*	
Serum albumin (g/dL), mean ± SD	3.29 ± 0.5
Total iron‐binding capacity (μg/dL), mean ± SD	210 ± 50
Malnutrition‐inflammation score (points), mean ± SD	5.7 ± 2.5
Interleukin‐6 (pg/mL), median (IQR)	9.23 (6.53, 21.09)

*Erythropoietin dose*	
Dose (Unit/week), median (IQR)	4000 (4000, 8000)
Erythropoietin resistance index (U/week/kg/g/dL), median (IQR)	8.28 (5.56, 14.18)

In Table 2, linear regression analyses showed that IL‐6 was positively associated with MIS (*β* = 0.684, 95% CI: 0.043–1.324, *p* = 0.037), and Kr was negatively associated with MIS (*β* = −0.585, 95% CI: −1.143 to −0.025, *p* = 0.040). ERI was not significantly associated with MIS (*p* = 0.313). When serum albumin was used as the outcome, Kr was positively associated (*β* = 0.152, 95% CI: 0.027–0.272, *p* = 0.018). IL‐6 and ERI were not significantly associated with serum albumin (*p* = 0.118 and *p* = 0.289, respectively).

**TABLE 2:** Associations of IL‐6, residual kidney clearance, and erythropoietin resistance index quartiles with continuous nutritional markers (malnutrition‐inflammation score and serum albumin).

Nutritional surrogate marker (outcome)	Determinant	Linear regression coefficient	95% confidence interval	*p* value
MIS score	IL‐6	0.684	0.043–1.324	0.037
	Kr	−0.585	−1.143–−0.025	0.040
	ERI	0.337	−0.328–1.002	0.313

Serum albumin	IL‐6	−0.118	−0.267–0.031	0.118
	Kr	0.152	0.027–0.272	0.018
	ERI	−0.081	−0.232–0.071	0.289

*Note:* MIS and serum albumin were analyzed as continuous outcome variables. IL‐6, ERI, and Kr were categorized into quartiles (Q1–Q4) and entered as predictors in linear regression analyses. Coefficients represent the change in MIS score or serum albumin level associated with each one‐quartile increase in the predictor variable.

In Table 3, in multivariable linear regression analyses, the associations between Kr quartile and both MIS and serum albumin remained significant after adjustment for age, sex, BMI, and total weekly Kt/V. In addition, the Spearman correlation in Table 4 showed that IL‐6 was significantly correlated with MIS (*r* = 0.390, 95% CI: 0.121–0.661, *p* = 0.004). IL‐6 was not significantly correlated with age, hemoglobin, creatinine, PTH, calcium, phosphate, PCR, or total Kt/V. A borderline correlation was observed between IL‐6 and serum albumin (*r* = −0.280, *p* = 0.062).

**TABLE 3:** Multivariable linear regression analyses of the associations between Kr quartile and nutritional markers.

Nutritional surrogate marker (outcome)	Determinant	Linear regression coefficient	95% confidence interval	*p* value
MIS	Unadjusted Kr	−0.585	−1.142–−0.028	0.040
	+ age	−0.642	−1.174–−1.111	0.019
	+ total Kt/V	−0.597	−1.161–−0.330	0.039
	+ BMI	−0.562	−1.124–−0.014	0.049
	+ gender	−0.589	−1.156–−0.224	0.042
	Full adjusted	−0.634	−1.186–−0.081	0.026

Albumin	Unadjusted Kr	0.152	0.027–0.277	0.018
	+ age	0.162	0.040–0.284	0.010
	+ total Kt/V	0.149	0.023–0.276	0.022
	+ BMI	0.151	0.024–0.278	0.021
	+ gender	0.153	0.026–0.280	0.020
	Full adjusted	0.160	0.032–0.288	0.015

*Note:* Kr quartile was modeled as an ordinal predictor. Sequential models were adjusted for age, sex, body mass index (BMI), and total weekly Kt/V as indicated. The fully adjusted model included age, sex, BMI, and total weekly Kt/V simultaneously. Coefficients represent the change in MIS score or serum albumin level associated with each one‐quartile increase in Kr.

**TABLE 4:** Factors affecting Interleukin‐6 level (IL‐6).

Parameters	Spearman Correlation Coefficient	95% CI (bootstrapping)	*p* value
Age	0.078	−0.266–0.422	0.656
Blood urea nitrogen	−0.174	−0.456–0.106	0.223
Hemoglobin	−0.122	−0.431–0.188	0.442
Creatinine	0.165	−0.149–0.480	0.302
Parathyroid hormone	−0.005	−0.331–0.321	0.976
Calcium	−0.027	−0.328–0.274	0.860
Phosphorus	−0.162	−0.328–0.274	0.276
Total weekly Kt/V	−0.188	−0.471–0.956	0.194
Protein catabolic rate	−0.122	−0.427–0.182	0.413
Normalized protein catabolic rate	−0.093	−0.412–0.225	0.566
Malnutrition‐inflammation score	0.390	0.121–0.661	0.004
Albumin	−0.280	−0.574–0.014	0.062
Total iron‐binding capacity	−0.243	−0.541–0.540	0.109

As shown in Table 5, ERI was not significantly correlated with age, IL‐6, MIS, serum albumin, or Kr. It was not significantly correlated with age (*r* = −0.108, *p* = 0.470), IL‐6 (*r* = 0.019, *p* = 0.899), MIS (*r* = 0.103, *p* = 0.442), serum albumin (*r* = 0.005, *p* = 0.971), or Kr (*r* = 0.015, *p* = 0.922).

**TABLE 5:** Factors affecting erythropoietin resistance index (ERI).

Parameters	Spearman Correlation Coefficient	95% CI (bootstrapping)	*p* value
Age	−0.108	−0.401–0.185	0.470
Blood urea nitrogen	0.265	−0.042–0.572	0.091
Creatinine	0.101	−0.204–0.406	0.516
Parathyroid hormone	−0.185	−0.470–0.100	0.204
Calcium	0.126	−0.162–0.414	0.393
Phosphorus	0.235	−0.054–0.524	0.111
Total weekly Kt/V	−0.094	−0.394–0.206	0.540
Residual kidney clearance: Kr	0.015	−0.278–0.307	0.922
Protein catabolic rate	0.065	−0.218–0.347	0.653
Normalized protein catabolic rate	0.071	−0.226–0.368	0.640
Albumin	0.005	−0.287–0.298	0.971
Total iron‐binding capacity	−0.149	−0.451–0.153	0.334
Interleukin‐6	0.019	−0.275–0.313	0.899
Malnutrition‐inflammation score	0.103	−0.160–0.366	0.442

In Table 6, Kr was negatively correlated with MIS (*r* = −0.358, 95% CI: −0.620 to −0.092, *p* = 0.008) and positively correlated with serum albumin (*r* = 0.343, 95% CI: 0.075–0.611, *p* = 0.012) and TIBC (*r* = 0.391, 95% CI: 0.139–0.643, *p* = 0.002). A borderline inverse correlation was noted between Kr and IL‐6 (*r* = −0.265, *p* = 0.057). Kr did not significantly correlate with age, hemoglobin, creatinine, or protein catabolic indices.

**TABLE 6:** Factors affecting residual kidney clearance (Kr).

Parameters	Spearman Correlation Coefficient	95% CI (bootstrapping)	*p* value
Age	0.041	−0.241–0.323	0.778
Blood urea nitrogen	0.226	−0.044–0.497	0.101
Hemoglobin	0.068	−0.239–0.376	0.663
Creatinine	−0.275	−0.566–0.167	0.065
Parathyroid hormone	−0.032	−0.294–0.230	0.810
Calcium	−0.331	−0.585–0.078	0.010
Phosphorus	0.118	−0.186–0.422	0.446
Protein catabolic rate	0.162	−0.146–0.470	0.304
Normalized protein catabolic rate	0.057	−0.244–0.358	0.711
Malnutrition‐inflammation score	−0.358	−0.620–−0.092	0.008
Albumin	0.343	0.075–0.611	0.012
Total iron‐binding capacity	0.391	0.139–0.643	0.002
Interleukin‐6	−0.265	−0.539–0.008	0.057

**FIGURE 1:**
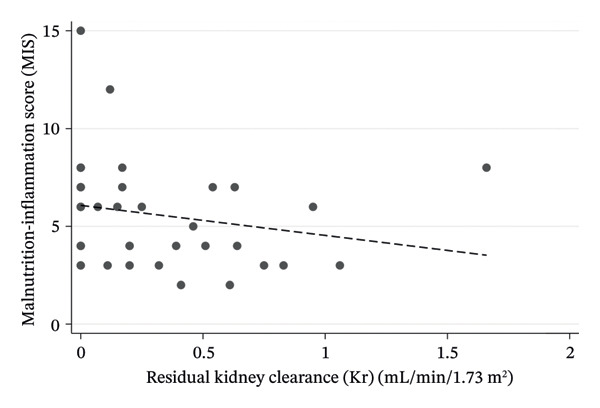
Scatterplot showing the relationship between residual kidney clearance (Kr) and malnutrition‐inflammation score (MIS).

**FIGURE 2:**
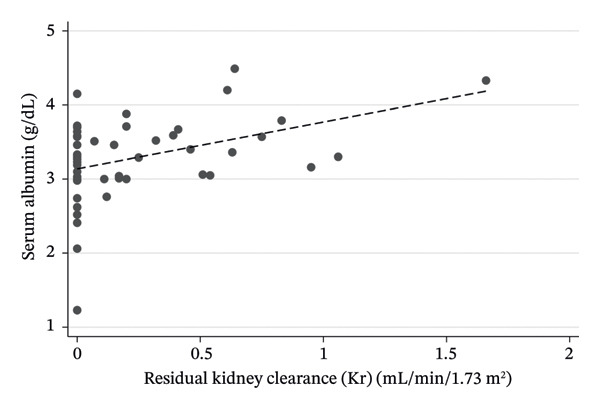
Scatterplot showing the relationship between residual kidney clearance (Kr) and serum albumin.

**FIGURE 3:**
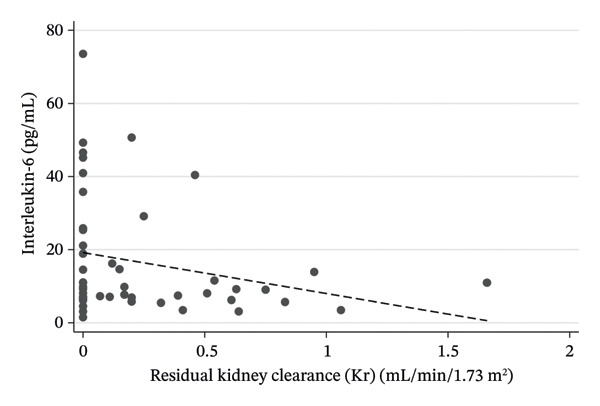
Scatterplot showing the relationship between residual kidney clearance (Kr) and Interleukin‐6 (IL‐6).

The relationships between Kr and key nutritional‐inflammatory markers are illustrated in Figures 1, 2, 3. Higher Kr was associated with lower MIS scores (Figure 1) and higher serum albumin levels (Figure 2). An inverse trend between Kr and IL‐6 was also observed (Figure 3), although the correlation did not reach statistical significance (*r* = −0.265, *p* = 0.057).

## 4. Discussion

This study provides additional insight into the interrelationship between nutritional status, inflammation, Kr, and erythropoietin resistance in patients undergoing PD, a population in which such associations have been far less explored compared to those on hemodialysis [29–35]. While previous studies have evaluated individual relationships between Kr, nutritional status, inflammation, and clinical outcomes, the combined assessment of MIS, serum albumin, IL‐6, erythropoietin resistance, and Kr within the same PD cohort remains limited. Notably, our primary finding was a significant positive correlation between the MIS and IL‐6, supporting the concept that malnutrition and systemic inflammation are tightly linked in ESKD. This finding is consistent with previous studies that have identified IL‐6 as a central mediator of inflammation in CKD and as a contributor to adverse outcomes, including poor nutritional status, cardiovascular disease, and anemia [5, 9, 23–27, 30, 31, 36–38].

In our cohort, more than half of the participants (57.5%) were at high risk of malnutrition (MIS ≥ 6), and 61.7% had serum albumin < 3.5 g/dL, reflecting the high prevalence of malnutrition in the PD population, consistent with prior reports in CKD and ESKD populations [3, 7]. The significant association between MIS and IL‐6 further supports the clinical utility of MIS as a composite measure of nutritional‐inflammatory burden in PD patients [9]. This is particularly relevant because chronic inflammation, as reflected by IL‐6, may amplify the effects of malnutrition and accelerate the development of complications, including anemia and cardiovascular disease [36–38]. Consistent with KDOQI guidelines, which recommend MIS as a validated assessment tool, our findings are consistent with its recommended use in identifying high‐risk patients and guiding nutritional management in the dialysis population [5]. In addition, an inverse association between Kr and MIS and IL‐6, along with a positive association with albumin, was observed, suggesting that better Kr may reflect a more favorable nutritional and inflammatory profile.

An additional observation from our study was the association between Kr and nutritional‐inflammation markers. Kr was inversely correlated with MIS and IL‐6 and positively associated with serum albumin levels. The observed association between Kr and nutritional‐inflammatory markers may reflect several biological pathways. Preserved Kr has been associated with enhanced removal of inflammatory mediators and uremic toxins, lower inflammatory burden, and improved volume control [14–17]. Kr may also contribute to the clearance of middle molecules and protein‐bound toxins that are incompletely removed by dialysis alone [14, 15]. Furthermore, better preservation of Kr may be associated with improved metabolic homeostasis, including maintenance of fluid, electrolyte, and acid–base balance [16–19], which may contribute to a more favorable nutritional and inflammatory profile. Conversely, there is evidence suggesting that systemic inflammation may adversely affect Kr. Proinflammatory cytokines and growth factors may contribute to progressive deterioration of kidney function through mechanisms involving endothelial dysfunction, oxidative stress, and direct tubular injury [39, 40]. Longitudinal studies have suggested a close relationship between Kr and systemic inflammation [41, 42]. Previous studies have associated preserved Kr with improved survival and quality of life in patients undergoing PD, but its relationship with nutritional and inflammatory status has received comparatively less attention [43]. Our findings are consistent with the International Society for Peritoneal Dialysis (ISPD) practice recommendations highlighting the importance of routinely monitoring and preserving Kr as part of a comprehensive approach to managing patients undergoing PD [19, 44].

In contrast, our secondary outcomes did not show significant correlations between MIS and ERI, nor between IL‐6 and ERI or albumin and ERI. These findings diverge from previous studies in hemodialysis populations that reported associations between malnutrition or inflammation and ESA responsiveness [33, 34, 45–48], suggesting that the metabolic and inflammatory profiles in PD patients may differ due to variations in ESA pharmacokinetics, Kr, or modality‐specific differences. Patients undergoing PD often retain greater Kr than those receiving maintenance hemodialysis, which may partially attenuate the relationship between inflammation and ESA responsiveness [13, 16, 17]. In addition, the relatively small sample size, restricted range of ERI values, and the inclusion criterion requiring stable ESA dosing may have limited the ability to detect more subtle associations. Moreover, the inflammatory burden in our cohort may have differed from that reported in previous hemodialysis studies [23–25, 45–48], potentially contributing to the discrepant findings. Taken together, these findings suggest that determinants of ESA responsiveness may differ between PD and hemodialysis populations.

The observed association between IL‐6 and MIS is consistent with the proposed role of IL‐6 as a marker of chronic inflammation in PD. As a cytokine with broad proinflammatory activity, IL‐6 has been associated with ESA resistance, cardiovascular disease, and PEW in previous studies [23, 24, 36–38, 45]. Although IL‐6 is not routinely measured in clinical practice due to cost and accessibility [49, 50], our findings suggest that IL‐6 may complement MIS in assessing nutritional‐inflammatory burden, especially when used alongside composite tools such as MIS. Together, these findings support the close relationship among inflammation, nutritional status, and Kr in patients undergoing PD.

This study has several limitations. Although the sample size was sufficient for the primary correlation analysis between MIS and IL‐6, the study was likely underpowered for exploratory analyses involving multiple nutritional and inflammatory markers simultaneously. Therefore, the negative findings, particularly regarding ERI, should be interpreted cautiously. All parameters were collected at a single time point, which may not fully reflect dynamic changes in nutritional or inflammatory status over time. Nonetheless, the use of standardized assessments, strict inclusion criteria, and a focus on a PD‐specific population, often underrepresented in comparative research, provide valuable context for interpreting these findings, and lay the groundwork for future prospective studies. Another limitation is the potential influence of unmeasured confounders. Factors such as dialysis vintage, comorbid conditions, and dietary intake were not comprehensively captured and may have influenced the observed associations. In addition, the small sample size limited the feasibility of multivariable adjustment.

## 5. Conclusion

MIS was significantly associated with IL‐6, highlighting the close relationship between malnutrition and systemic inflammation in patients undergoing PD. Higher Kr was associated with a more favorable nutritional‐inflammatory profile, whereas ERI was not significantly associated with nutritional or inflammatory markers.

## Author Contributions

W.J. was responsible for data interpretation, data cleaning, statistical analysis, and writing manuscript. C.W. was responsible for the data acquisition, asking for informed consent, drafting of work, and writing manuscript. T.B. and S.W. were responsible for data acquisition and asking for informed consent. A.C. was responsible for design of the study; drafting of work; data acquisition, cleaning, and interpretation; statistical analysis; writing manuscript; and primary corresponding author.

## Funding

No funding was received for this manuscript.

## Disclosure

All authors contributed to the work, as well as reading and approving the final manuscript.

## Conflicts of Interest

The authors declare no conflicts of interest.

## Data Availability

The data that support the findings of this study are available upon request from the corresponding author. The data are not publicly available due to privacy or ethical restrictions.

## References

[1] Kovesdy C. P. , Epidemiology of Chronic Kidney Disease: An Update 2022, Kidney International Supplements. (April 2022) 12, no. 1, 7–11, 10.1016/j.kisu.2021.11.003. 35529086 PMC9073222

[2] Zhang S. , Ren H. F. , Du R. X. , Sun W. L. , Fu M. L. , and Zhang X. C. , Global, Regional, and National Burden of Kidney Dysfunction From 1990 to 2019: A Systematic Analysis From the Global Burden of Disease Study 2019, BMC Public Health. (June 2023) 23, no. 1, 10.1186/s12889-023-16130-8. PMC10288715 37353821

[3] Zha Y. and Qian Q. , Protein Nutrition and Malnutrition in CKD and ESRD, Nutrients. (February 2017) 9, no. 3, 10.3390/nu9030208. PMC5372871 28264439

[4] Nascimento M. M. , Pecoits-Filho R. , Lindholm B. , Riella M. C. , and Stenvinkel P. , Inflammation, Malnutrition and Atherosclerosis in End-Stage Renal Disease: A Global Perspective, Blood Purification. (2002) 20, no. 5, 454–458, 10.1159/000063559. 12207091

[5] Ikizler T. A. , Burrowes J. D. , Byham-Gray L. D. et al., KDOQI Clinical Practice Guideline for Nutrition in CKD: 2020 Update, American Journal of Kidney Diseases. (September 2020) 76, no. 3, S1–S107, 10.1053/j.ajkd.2020.05.006. 32829751

[6] Kittiskulnam P. , Chuengsaman P. , Kanjanabuch T. et al., Protein-Energy Wasting and Mortality Risk Prediction Among Peritoneal Dialysis Patients, Journal of Renal Nutrition. (November 2021) 31, no. 6, 679–686, 10.1053/j.jrn.2020.11.007. 33642190

[7] Chung S. , Koh E. S. , Shin S. J. , and Park C. W. , Malnutrition in Patients With Chronic Kidney Disease, Open Journal of Internal Medicine. (2012) 02, no. 02, 89–99, 10.4236/ojim.2012.22018.

[8] Zhong J. , Fan C. , Li L. et al., Association Between Dietary Inflammation Index and Malnutrition Status in Peritoneal Dialysis Patients: A Cross-Sectional Study, Frontiers in Nutrition. (January 2026) 12, 10.3389/fnut.2025.1664787. PMC12886001 41674797

[9] Kalantar-Zadeh K. , Kopple J. D. , Block G. , and Humphreys M. H. , A Malnutrition-Inflammation Score is Correlated With Morbidity and Mortality in Maintenance Hemodialysis Patients, American Journal of Kidney Diseases. (December 2001) 38, no. 6, 1251–1263, 10.1053/ajkd.2001.29222. 11728958

[10] Chertow G. M. , Chang A. M. , Felker G. M. et al., IL-6 Inhibition With Clazakizumab in Patients Receiving Maintenance Dialysis: A Randomized Phase 2b Trial, Nature Medicine. (Auguest 2024) 30, no. 8, 2328–2336, 10.1038/s41591-024-03043-1. PMC11333272 38796655

[11] Elphick E. H. , Zavvos V. , Belcher J. et al., The Role of Peritoneal Interleukin-6 in Predicting Patient Survival on Peritoneal Dialysis, Kidney International Reports. (September 2025) 10, no. 9, 3164–3173, 10.1016/j.ekir.2025.06.049. 40980662 PMC12446933

[12] Pecoits-Filho R. and Medina E. , The Body Speaks Louder Than the Belly: Systemic Inflammation Drives Peritoneal Dialysis Outcomes, Kidney International Reports. (September 2025) 10, no. 9, 2907–2908, 10.1016/j.ekir.2025.06.051. 40980672 PMC12446945

[13] Vilar E. , Wellsted D. , Chandna S. M. , Greenwood R. N. , and Farrington K. , Residual Renal Function Improves Outcome in Incremental Haemodialysis Despite Reduced Dialysis Dose, Nephrology Dialysis Transplantation. (Auguest 2009) 24, no. 8, 2502–2510, 10.1093/ndt/gfp071. 19240122

[14] Fry A. C. , Singh D. K. , Chandna S. M. , and Farrington K. , Relative Importance of Residual Renal Function and Convection in Determining Beta-2-Microglobulin Levels in High-Flux Haemodialysis and On-Line Haemodiafiltration, Blood Purification. (2007) 25, no. 3, 295–302, 10.1159/000104870. 17622712

[15] Shafi T. , Jaar B. G. , Plantinga L. C. et al., Association of Residual Urine Output With Mortality, Quality of Life, and Inflammation in Incident Hemodialysis Patients: The Choices for Healthy Outcomes in Caring for End-Stage Renal Disease (CHOICE) Study, American Journal of Kidney Diseases. (Auguest 2010) 56, no. 2, 348–358, 10.1053/j.ajkd.2010.03.020. 20605303 PMC2910835

[16] Mangalgi S. , Joshi V. , Misra M. , and Chaudhary K. , Residual Kidney Function and the Impact of Dialysis Modality, Kidney and Dialysis. (September 2025) 5, no. 3, 10.3390/kidneydial5030043.

[17] Obi Y. , Raimann J. G. , Kalantar-Zadeh K. , and Murea M. , Residual Kidney Function in Hemodialysis: Its Importance and Contribution to Improved Patient Outcomes, Toxins. (June 2024) 16, no. 7, 10.3390/toxins16070298. PMC11281084 39057938

[18] Boudville N. and De Moraes T. P. , 2005 Guidelines on Targets for Solute and Fluid Removal in Adults Being Treated With Chronic Peritoneal Dialysis: 2019 Update of the Literature and Revision of Recommendations, Peritoneal Dialysis International. (May 2020) 40, no. 3, 254–260, 10.1177/0896860819898307. 32048566

[19] Wang A. Y. M. , Brimble K. S. , Brunier G. et al., ISPD Cardiovascular and Metabolic Guidelines in Adult Peritoneal Dialysis Patients Part I—Assessment and Management of Various Cardiovascular Risk Factors, Peritoneal Dialysis International. (July 2015) 35, no. 4, 379–387, 10.3747/pdi.2014.00279. 26228782 PMC4520720

[20] Portolés J. , Martín L. , Broseta J. J. , and Cases A. , Anemia in Chronic Kidney Disease: From Pathophysiology and Current Treatments, to Future Agents, Frontiers of Medicine. (March 2021) 8, 10.3389/fmed.2021.642296. PMC8032930 33842503

[21] Del Vecchio L. , Pozzoni P. , Andrulli S. , and Locatelli F. , Inflammation and Resistance to Treatment With Recombinant Human Erythropoietin, Journal of Renal Nutrition. (January 2005) 15, no. 1, 137–141, 10.1053/j.jrn.2004.09.024. 15648023

[22] Santos E. J. F. , Dias R. S. C. , Lima J. F. D. B. , Salgado Filho N. , and Miranda Dos Santos A. , Erythropoietin Resistance in Patients With Chronic Kidney Disease: Current Perspectives, International Journal of Novel Reaserch and Development. (October 2020) 13, 231–237, 10.2147/IJNRD.S239151. PMC7549651 33116754

[23] Akchurin O. , Patino E. , Dalal V. et al., Interleukin-6 Contributes to the Development of Anemia in Juvenile CKD, Kidney International Reports. (March 2019) 4, no. 3, 470–483, 10.1016/j.ekir.2018.12.006. 30899874 PMC6409399

[24] Coyne D. W. and Fleming R. , Will Targeting Interleukin-6 in the Anemia of CKD Change Our Treatment Paradigm?, Journal of the American Society of Nephrology. (January 2021) 32, no. 1, 6–8, 10.1681/ASN.2020101476. 33272966 PMC7894666

[25] Won H. S. , Kim H. G. , Yun Y. S. et al., IL 6 is an Independent Risk Factor for Resistance to Erythropoiesis‐Stimulating Agents in Erythropoiesis‐Patients Without Iron Deficiency, Hemodialysis International. (January 2012) 16, no. 1, 31–37, 10.1111/j.1542-4758.2011.00635.x. 22284696

[26] Foley R. N. , Anaemia: Cardiovascular Adaptations and Maladaptive Responses in Chronic Kidney Disease, Nephrology Dialysis Transplantation. (November 2002) 17, no. suppl 11, 32–34, 10.1093/ndt/17.suppl_11.32. 12386255

[27] Zhao X. , Gan L. , Hou F. F. et al., The Influencing Factors of the Erythropoietin Resistance Index and its Association With All-Cause Mortality in Maintenance Hemodialysis Patients, Renal Failure. (December 2024) 46, no. 1, 10.1080/0886022X.2023.2290922. PMC10798285 38234178

[28] Von E. E. , Altman D. G. , Egger M. , Pocock S. J. , Gøtzsche P. C. , and Vandenbroucke J. P. , The Strengthening the Reporting of Observational Studies in Epidemiology (STROBE) Statement: Guidelines for Reporting Observational Studies, International Journal of Surgery. (December 2014) 12, no. 12, 1495–1499, 10.1016/j.ijsu.2014.07.013. 25046131

[29] Abe M. , Okada K. , Maruyama T. , Maruyama N. , Matsumoto K. , and Soma M. , Relationship Between Erythropoietin Responsiveness, Insulin Resistance, and Malnutrition-Inflammation-Atherosclerosis (Mia) Syndrome in Hemodialysis Patients With Diabetes, International Journal of Artificial Organs. (January 2011) 34, no. 1, 16–25, 10.5301/IJAO.2011.6314. 21298620

[30] Feret W. , Safranow K. , Kwiatkowska E. , Daniel A. , and Ciechanowski K. , Malnutrition and Erythropoietin Resistance Among Patients With End-Stage Kidney Disease: Where is the Perpetrator of Disaster?, Nutrients. (December 2022) 14, no. 24, 10.3390/nu14245318. PMC9787334 36558477

[31] De M. R. , Grootendorst D. C. , Indemans F. , Boeschoten E. W. , Krediet R. T. , and Dekker F. W. , Association Between Serum Albumin and Mortality in Dialysis Patients is Partly Explained by Inflammation, and Not by Malnutrition, Journal of Renal Nutrition. (March 2009) 19, no. 2, 127–135, 10.1053/j.jrn.2008.08.003. 19218039

[32] Feret W. , Safranow K. , Ciechanowski K. , and Kwiatkowska E. , How is Body Composition and Nutrition Status Associated With Erythropoietin Response in Hemodialyzed Patients? A Single-Center Prospective Cohort Study, JCM. (April 2022) 11, no. 9, 10.3390/jcm11092426. PMC9105329 35566552

[33] Okazaki M. , Komatsu M. , Shiohira S. et al., Associations Between the Erythropoiesis-Stimulating Agent Resistance Index and the Geriatric Nutritional Risk Index of Maintenance Hemodialysis Patients and Increased Mortality, Renal Replacement Therapy. (December 2015) 1, no. 1, 10.1186/s41100-015-0002-2.

[34] Kim D. H. , Shin J. , and Oh D. J. , Hemoglobin Variability is Associated With Nutritional Status in Hemodialysis Patients Undergoing Darbepoetin-Alfa Treatment.PDF, 10.1093/ndt/gfad063c_2621.

[35] Don B. R. and Kaysen G. , Serum Albumin: Relationship to Inflammation and Nutrition, Seminars in Dialysis. (November 2004) 17, no. 6, 432–437, 10.1111/j.0894-0959.2004.17603.x. 15660573

[36] Jones S. A. , Fraser D. J. , Fielding C. A. , and Jones G. W. , Interleukin-6 in Renal Disease and Therapy, Nephrology Dialysis Transplantation. (April 2015) 30, no. 4, 564–574, 10.1093/ndt/gfu233. 25011387

[37] Graterol Torres F. , Molina M. , Soler-Majoral J. et al., Evolving Concepts on Inflammatory Biomarkers and Malnutrition in Chronic Kidney Disease, Nutrients. (October 2022) 14, no. 20, 10.3390/nu14204297. PMC9611115 36296981

[38] Akchurin O. M. and Kaskel F. , Update on Inflammation in Chronic Kidney Disease, Blood Purification. (2015) 39, no. 1–3, 84–92, 10.1159/000368940. 25662331

[39] Stompór T. , Zdzienicka A. , Motyka M. , Dembińska-Kieć A. , Davies S. J. , and Sulowicz W. , Selected Growth Factors in Peritoneal Dialysis: Their Relationship to Markers of Inflammation, Dialysis Adequacy, Residual Renal Function, and Peritoneal Membrane Transport, Peritoneal Dialysis International. (2002) 22, no. 6, 670–676, 10.1177/089686080202200605. 12556068

[40] Gonçalves G. T. , Santos L. M. D. M. , Figueiredo P. H. S. et al., Lu T. L. , High Level of Soluble Tumor Necrosis Factor Receptors is Associated With Lower Residual Diuresis Volume in Patients on Hemodialysis: An Exploratory Study, PLoS One. (April 2025) 20, no. 4, 10.1371/journal.pone.0320019. PMC12040094 40299805

[41] Szeto C. C. , Kwan B. C. H. , Chow K. M. et al., Eller K. , The Effect of Neutral Peritoneal Dialysis Solution With Low Glucose-Degradation-Product on the Fluid Status and Body Composition–A Randomized Control Trial, PLoS One. (October 2015) 10, no. 10, 10.1371/journal.pone.0141425. PMC4625015 26510186

[42] Martín‐Del‐Campo F. , González‐Espinoza L. , Rojas‐Campos E. et al., Conventional Nutritional Counselling Maintains Nutritional Status of Patients on Continuous Ambulatory Peritoneal Dialysis in Spite of Systemic Inflammation and Decrease of Residual Renal Function, Nephrology. (Auguest 2009) 14, no. 5, 493–498, 10.1111/j.1440-1797.2008.01081.x. 19674317

[43] Adequacy of Dialysis and Nutrition in Continuous Peritoneal Dialysis: Association With Clinical Outcomes. Canada-USA (CANUSA) Peritoneal Dialysis Study Group, Journal of the American Society of Nephrology. (February 1996) 7, no. 2, 198–207, 10.1681/ASN.V72198. 8785388

[44] Brown E. A. , Blake P. G. , Boudville N. et al., International Society for Peritoneal Dialysis Practice Recommendations: Prescribing High-Quality Goal-Directed Peritoneal Dialysis, Peritoneal Dialysis International. (May 2020) 40, no. 3, 244–253, 10.1177/0896860819895364. 32063219

[45] Sánchez Hernández K. V. , Larrosa-Haro A. , González Hita M. et al., Interleukine 6 and C-Reactive Protein Predict Growth Impairment and Acute Malnutrition in Children and Adolescents With Chronic Kidney Disease, Nutricion Hospitalaria. (2019) 10.20960/nh.02640. 31657603

[46] Akgul A. , Bilgic A. , Sezer S. et al., Effect of Protein‐Energy Malnutrition on Erythropoietin Requirement in Maintenance Hemodialysis Patients, Hemodialysis International. (April 2007) 11, no. 2, 198–203, 10.1111/j.1542-4758.2007.00169.x. 17403171

[47] Neves P. L. , Morgado E. , Faísca M. , Carrasqueira H. , Baptista A. , and Silva A. P. , Nutritional and Inflammatory Status Influence Darbepoetin Dose in Pre-Dialysis Elderly Patients, International Urology and Nephrology. (February 2007) 38, no. 3–4, 811–813, 10.1007/s11255-006-0077-3. 17160447

[48] Locatelli F. , Andrulli S. , Memoli B. et al., Nutritional-Inflammation Status and Resistance to Erythropoietin Therapy in Haemodialysis Patients, Nephrology Dialysis Transplantation. (April 2006) 21, no. 4, 991–998, 10.1093/ndt/gfk011. 16384825

[49] Shioya M. , Yoshida T. , Kasai K. et al., Inflammatory Factors for Hypoalbuminemia in J Apanese Peritoneal Dialysis Patients, Nephrology. (Auguest 2013) 18, no. 8, 539–544, 10.1111/nep.12106. 23718260

[50] Malho Guedes A. , Calças Marques R. , Ribeiro B. et al., Peritoneal Protein Loss, Inflammation, and Nutrition: Refuting Myths, Frontiers of Medicine. (May 2022) 9, 10.3389/fmed.2022.884061. PMC9178188 35692552

